# Use of wearable devices for post-discharge monitoring of ICU patients: a feasibility study

**DOI:** 10.1186/s40560-017-0261-9

**Published:** 2017-11-21

**Authors:** Ryan R. Kroll, Erica D. McKenzie, J. Gordon Boyd, Prameet Sheth, Daniel Howes, Michael Wood, David M. Maslove, for the WEARable Information Technology for hospital INpatients (WEARIT-IN) study group

**Affiliations:** 1 Department of Critical Care Medicine, Queen’s University and Kingston Health Sciences Centre, Kingston, Ontario Canada; 2 School of Medicine, Queen’s University, Kingston, Ontario Canada; 3 Department of Medicine, Queen’s University and Kingston Health Sciences Centre, Kingston, Ontario Canada; 4 Department of Pathology and Molecular Medicine, Queen’s University and Health Sciences Centre, Kingston, Ontario Canada; 5 Department of Emergency Medicine, Queen’s University and Kingston Health Sciences Centre, Kingston, Ontario Canada; 6 Department of Neuroscience, Queen’s University, Kingston, Ontario Canada; 7 Kingston Health Sciences Centre, Kingston General Hospital, Davies 2, 76 Stuart St., Kingston, Ontario K7L 2V7 Canada

**Keywords:** Wearable devices, Medical informatics, Mobile health technologies, Validation study, Critical care, Sleep quality, Heart rate monitoring

## Abstract

**Background:**

Wearable devices generate signals detecting activity, sleep, and heart rate, all of which could enable detailed and near-continuous characterization of recovery following critical illness.

**Methods:**

To determine the feasibility of using a wrist-worn personal fitness tracker among patients recovering from critical illness, we conducted a prospective observational study of a convenience sample of 50 stable ICU patients. We assessed device wearability, the extent of data capture, sensitivity and specificity for detecting heart rate excursions, and correlations with questionnaire-derived sleep quality measures.

**Results:**

Wearable devices were worn over a 24-h period, with excellent capture of data. While specificity for the detection of tachycardia was high (98.8%), sensitivity was low to moderate (69.5%). There was a moderate correlation between wearable-derived sleep duration and questionnaire-derived sleep quality (*r* = 0.33, *P* = 0.03). Devices were well-tolerated and demonstrated no degradation in quality of data acquisition over time.

**Conclusions:**

We found that wearable devices could be worn by patients recovering from critical illness and could generate useful data for the majority of patients with little adverse effect. Further development and study are needed to better define and enhance the role of wearables in the monitoring of post-ICU recovery.

**Trial registration:**

Clinicaltrials.gov, NCT02527408

**Electronic supplementary material:**

The online version of this article (10.1186/s40560-017-0261-9) contains supplementary material, which is available to authorized users.

## Background

Consumer interest in personal health tracking has recently increased, leading to an industry in wearable devices now valued at more than $9 billion worldwide [1]. With more wearables in use than ever before, there has been growing enthusiasm for their potential to improve health care delivery [2]. Current clinical uses for wearable devices are mostly limited to outpatient settings, with a focus on the management of chronic diseases [3–5]. Newer generation devices generate data that could also be useful in characterizing convalescence from acute illness. These include photoplethysmography (PPG) sensors to detect heart rate [6, 7], as well as accelerometers to track activity and movement [3, 8, 9].

Frequent heart rate tracking has the potential to identify episodes of clinical deterioration early. Accelerometer data could potentially be used to encourage mobilization, objectively measure functional status, and track progress towards rehabilitation goals. Wrist-worn accelerometers have also been used to evaluate sleep quality in healthy subjects [10, 11]. In the inpatient and intensive care unit (ICU) settings, where poor sleep has been linked with adverse outcomes [12, 13], data describing sleep quality may be useful in identifying targets for sleep-promoting interventions [14].

There is little clinical evidence to inform the practice of using wearables in health care, most of which is focused on chronic conditions. Newer consumer-grade wearables have been evaluated in only a handful of studies examining their accuracy among healthy volunteers [3–5]. These studies have called for evaluations of this technology among a wider range of patient populations.

In this study, we examine the feasibility of using a common consumer-grade wearable device to monitor patients recovering from critical illness. We enrolled patients who no longer required intensive care measures but remained in the ICU prior to ward transfer, in order to best approximate post-ICU settings like the general wards, while still collecting gold standard data to validate device functionality. We report on a number of practical considerations that could affect the deployment of wearables including overall wearability, completeness of data capture, device longevity, and risk of transmitting nosocomial infections. We also evaluated the accuracy of wearables for measuring sleep quality and identifying changes in heart rate that might be clinically relevant. We hypothesized that patients recovering from critical illness would be able to wear wrist-worn devices and that useful data could be collected from these with a moderate degree of accuracy.

## Methods

### Patients and setting

This prospective observational study was conducted in a 33-bed general medical-surgical/trauma ICU in southeastern Ontario, between August 2015 and February 2016. Adult patients (age > 17) were included if they were receiving continuous cardiac and oxygen saturation monitoring, but were otherwise receiving ward-level treatment. Exclusion criteria included mechanical ventilation, vasopressor support, and continuous sedation or analgesia. We specifically chose to study patients who were still in the ICU, as this was the most practical way to obtain gold standard measurements of heart rate and sleep quality, which would otherwise require the use of Holter monitors and complex follow-up procedures. To reduce the potential risk of transmitting nosocomial infections, patients under contact precautions for methicillin-resistant *Staphylococcus aureus* (MRSA) and *Clostridium difficile* infections were also excluded. We also excluded patients at risk of vascular compromise of the arm on which the wearable device was to be placed, such as patients with upper extremity deep venous thrombosis, peripherally inserted central catheters, radial arterial lines, dialysis fistulas, and severe upper extremity trauma. As this was a feasibility study, a convenience sample of 50 participants was recruited.

### Ethics, consent, and permissions

All participating patients, or substitute decision makers on their behalf, provided written informed consent for participation in this study. The Health Sciences Research Ethics Board at Queen’s University reviewed and approved the study protocol (DMED-1818-15), and the trial was registered with clinicaltrials.gov (NCT02527408).

### Device

**Fig. 1:**
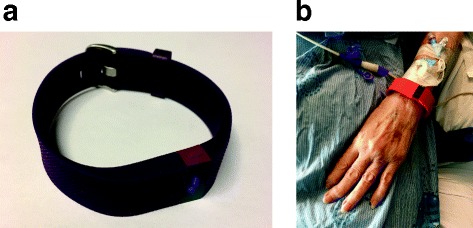
The Fitbit Charge HR device used in the study (**a**). The wearable device as worn by a patient on the inpatient ward following ICU discharge (**b**)

Participating patients wore the Fitbit Charge HR device (Fitbit, San Francisco, CA, USA) for a single 24-h period (Fig. 1). The Fitbit Charge HR is a commercially available wrist-worn wearable that records heart rate, steps, and sleep quality. The study employed three size large wearable devices (15.7 to 19.3 cm wrist circumference) and three size extra-large wearable devices (19.6 to 22.6 cm wrist circumference). In an effort to reduce the risk of potential iatrogenic infection, we used disinfectant wipes to thoroughly clean wearables between uses. All devices were applied to participants by a study investigator or coordinator.

### Data monitoring and capture

We used continuous pulse oximetry pulse rate recordings (SPO2-R) as a comparison measure of heart rate (HR) in order to evaluate the ability of wearables to detect both tachycardia (HR > 100 bpm) and bradycardia (HR < 50 bpm). We used SPO2-R values as a comparator as both SPO2-R and wearable device values reflect the pulse rate (rather than electrical heart rate), and because this is a widely accepted method of heart rate measurement. The wearables recorded heart rate values every 5 min, while the SPO2-R recorded heart rate values every minute. Cardiac rhythm was assessed at the time of device application, and again at the time of removal, at which time data regarding sleep quality was also collected using the Richards-Campbell Sleep Questionnaire (RCSQ) [15]. This survey uses a visual analog scale to assess sleep depth, latency, awakenings, percentage of time awake, and overall quality of sleep. The RCSQ was completed either by the patients themselves or by their designated night shift nurse, a practice previously shown to have slight to moderate agreement with self-assessment [16]. Due to the interaction between sleep and delirium in the ICU [17], patients were screened for delirium by a trained researcher using the confusion assessment method (CAM)-ICU at the time of device application, and again at the time of device removal.

Wearable-reported sleep data included time of sleep onset and awakening, sleep duration, minutes asleep, minutes awake, restless count, and a calculated measure of sleep quality. Overall sleep quality was taken as the average across sleep episodes, weighted by the duration of each sleep episode. The percentage of total sleep occurring during nighttime hours, which we defined as 22:00 to 06:00, and the percentage of nighttime hours spent asleep were calculated. For participants who had no Fitbit-detected sleep over the recording period, a score of 0 was given for all sleep parameters. Methods for obtaining wearable and SPO2-R data are reported elsewhere [18], and in the Supplementary Content (see Additional file 1).

### Microbiological assessment

We conducted microbiologic sampling of the wearables used from a convenience subset of patients (*n* = 16) in order to evaluate both the risk of transmitting nosocomial pathogens from repeated application of wearables to different patients, as well as the efficacy of our disinfection practices (see Additional file 1).

### Statistical analysis

In the absence of preliminary data to inform a sample size calculation, we targeted an enrollment of 50 patients, a cohort size equal to that used in a similar study of wrist-worn wearables for heart rate tracking in healthy volunteers [5]. In addition to basic descriptive statistics, we calculated the sensitivity and specificity of the wearables for detecting tachycardia and bradycardia. Based on the PPG mechanism of heart rate sensing employed in consumer-grade wearables, we hypothesized that the accuracy of wearable device heart rate tracking may be different in patients not in sinus rhythm and further analyzed these patients as a subgroup. We calculated Pearson correlation coefficients between the various wearable-derived measures of sleep quality and the RCSQ measures of sleep quality. Based on the mechanism of sleep sensing, which relies on the absence of movement, we hypothesized that the accuracy of wearables for sleep tracking may differ in patients with delirium, and further analyzed these patients as a subgroup. Statistical analyses for this study were performed using R (v 3.2.2).

## Results

### Patients and device wearability

We enrolled a total of 50 patients between August 2015 and January 2016 (Table 1). The median wrist circumference in our cohort was 18.6 cm (SD 1.9 cm), with 6 of the 50 patients enrolled having moderate or severe edema of the wrist at the time of device application. The size large device was used for 23 patients (46%), while the size extra-large was used for 27 patients (54%). While there were no patients for whom the wearable device could not be fitted, the fit was noted to be very tight in one patient, and very loose in two patients. Devices were adjusted only once at the time of application and were not re-assessed by study personnel for the duration of the 24-h recording period. No intravenous lines were re-sited in order to facilitate application, although hospital identification wristbands had to be relocated in some cases. No wearables required removal during the monitoring period as a result of patient discomfort. The wearable device was removed prior to the completion of the monitoring period in two patients; one patient was discharged earlier than expected from the ICU, while another developed a diffuse drug-associated rash. Excluding patients whose devices were removed early, the devices were unable to detect a heart rate reading 4% of the time.

**Table 1:** Characteristics of patients included in the study (*n* = 50)

Mean heart rate (bpm)	88.3	
Mean age (years)	64	
	Patients (*n* = 50)	%
Male	26	52
Female	24	48
Admission diagnosis		
Respiratory	12	24
Sepsis	7	14
Surgical	7	14
Neurologic	11	22
Trauma	3	6
Cardiovascular	6	12
Medical	4	8
Sinus rhythm		
At start of monitoring	43	86
At end of monitoring	42	84
Personal fitness tracker size used		
Large	23	46
Extra large	27	54

### Tachycardia and bradycardia detection

**Fig. 2:**
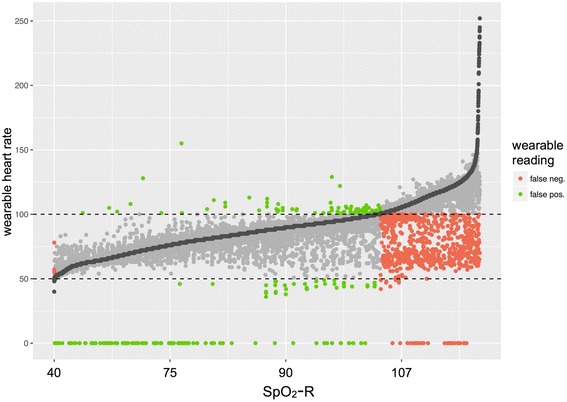
Accuracy of wearable-derived heart rates for the detection of tachycardia (HR > 100) or bradycardia (HR < 50) as determined by SPO2 heart rates. The SPO2-derived values (dark gray) are shown sorted from lowest to highest heart rate. The corresponding wearable-derived heart rate is shown in either light gray (correct classification), green (false positive), or red (false negative). The majority of misclassified heart rates are false negatives for the detection of tachycardia. Some misclassification is due to wearable device readings of “0,” reflecting data not captured by the device

We identified 13 SPO2-R-confirmed readings of bradycardia among four patients, all of whom were in sinus rhythm. Further statistical analysis was not done due to this small sample. The wearable had a sensitivity of 69.5% and specificity of 98.8% for the detection of tachycardia (Table 2 and Fig. 2). Among patients not in sinus rhythm (*n* = 8), the specificity for detecting tachycardia was similar (99.5%), although sensitivity was worse (51.6%). For faster heart rates (> 150 bpm), wearable device concordance with SPO2-R was poor. However, in many such cases, the wearable device reading showed better agreement with the true heart rate measured by continuous ECG, than did the SPO2-R readings, which tended to be falsely high.

**Table 2:** Test performance characteristics for personal fitness tracker detection of tachycardia, as compared to SPO2-R

	Sinus rhythm	Atrial fibrillation
Sensitivity	0.695	0.516
Specificity	0.988	0.995
Positive predictive value	0.948	0.983
Negative predictive value	0.914	0.804
Accuracy	0.92	0.836

### Sleep data

**Fig. 3:**
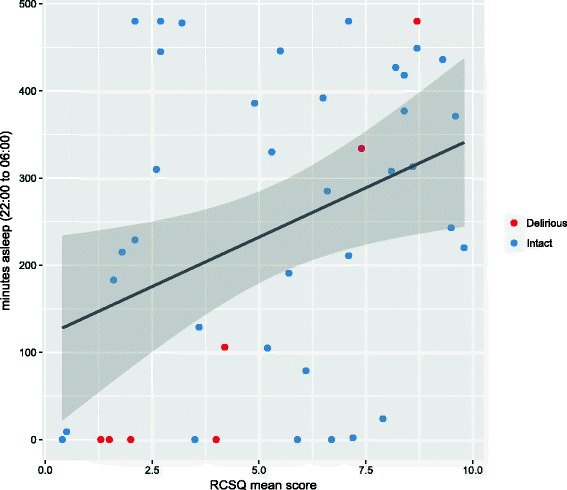
Correlation between mean score on the Richards-Campbell Sleep Questionnaire (RCSQ) and wearable-derived measure of the number of minutes asleep overnight (between 22:00 and 06:00). The Pearson correlation coefficient was 0.33 (95% CI 0.04 - 0.58)

A summary of the sleep quality data collected by the wearables is shown in Table 3. Among the 47 participants who had complete wearable sleep data recorded, the median wearable-reported sleep duration was 6.6 h (interquartile range [IQR] 2.7–13.5 h) and the median number of sleep periods was 2 (IQR 1–4). Five participants (11%) had no wearable device documented sleep for the entirety of the 24-h monitoring period. Among the 43 participants for whom the RCSQ was completed, the median total score was 5.7/10.0 (IQR 2.7–8.0/10.0). There was a moderate correlation between wearable-derived sleep duration and total RCSQ score (*r* = 0.33, *P* = 0.03, 95% confidence interval [CI] 0.04, 0.58) (Fig. 3). The correlation between the percentage of nighttime asleep, as reported by the wearable device, and total RCSQ score was 0.36 (*P* = 0.02, 95% CI 0.07, 0.60). The correlation between the Fitbit-reported number of sleep periods and RCSQ-reported awakenings was 0.38 (*P* = 0.01, 95% CI 0.09, 0.61). There were no significant differences in wearable-reported sleep parameters between the CAM-ICU positive (*n* = 8) and CAM-ICU negative participants; however, 25% of CAM-ICU positive participants recorded no sleep over the entire 24-h monitoring period, compared to 8% of CAM-ICU negative participants.

**Table 3:** Summary of wearable-reported and RCSQ sleep parameters

	Median	(IQR)
Wearable		
Total sleep duration, hours	6.6	(2.7–13.5)
Asleep time, hours	6.1	(2.6–12.5)
Restless count	7	(2.5–19.0)
Sleep quality A	45.8	(38.0–63.5)
# Sleep periods	2	(1.0–4.0)
22:00–6:00 sleep as % of total	50%	(15–80%)
% of 22:00–6:00 asleep	48%	(3–84%)
RCSQ		
Mean score	5.7	(2.7–8.0)
1. Sleep depth	5	(3.2–7.6)
2. Sleep latency	6.2	(2.7–8.9)
3. Awakening	5	(2.6–8.6)
4. Returning to sleep	6.4	(2.1–9.1)
5. Sleep quality	5.7	(1.6–8.6)

*RCSQ* Richards-Campbell Sleep Questionnaire

### Device reusability

Wearables were not found to be a significant source of pathogenic bacteria. Microbiologic sampling revealed bacteria consistent with commensal skin flora (*Staphylococcus epidermidis*) and/or environmental organisms (*Bacillus* species). *S. epidermidis* was only observed in samples taken prior to hydrogen peroxide disinfection, while *Bacillus* species were found in both pre- and post-disinfection specimens. Individual wearable devices were used between 5 and 13 times. There were no differences in wearable-SPO2-R heart rate correlations between the first and second half of the study (*P* = 0.18).

## Discussion

The long-term adverse consequences of critical illness are increasingly being recognized as a research priority in critical care [19]. A growing body of research is now examining the determinants and potential modifiers of post-ICU recovery, including at least one study that made use of a wearable device to track patient movement and activity [20]. However, post-ICU recovery research currently lacks the richness of data available to researchers focused on the ICU stay itself since post-discharge data collection is limited to infrequent visits to follow-up clinics, or in many cases is nonexistent. New strategies are needed to collect data—ideally on a continuous basis—that better describes ICU recovery on the wards and in the patient’s home environment.

To this end, we undertook an observational study to determine the feasibility of using a commercial-grade wearable device to monitor recovery after critical illness. Overall, the device was well tolerated and captured the vast majority of available data. For the detection of tachycardia, we found the wearable delivered high specificity and positive predictive value, but only low to moderate sensitivity. Much of the undercounting of fast heart rates by the wearable device was seen in patients who were not in sinus rhythm during at least some portion of the monitoring period. Compared to a validated sleep questionnaire, the wearable device had a moderate correlation with several metrics of sleep quality. Device performance did not appear to degrade over time. The wearables studied did not appear to be a significant source of nosocomial pathogens, although the presence of *Bacillus* species even after device cleaning suggests that spore-forming organisms could persist on some devices. Whether or not wearables would have to be reused at all would depend on their costs—which currently are relatively low—compared to the potential cost savings achieved with better clinical outcomes. The use of wearables to monitor convalescence after ICU discharge will ultimately pertain to patients who no longer require the resources of heavily monitored settings. To that end, our results are generalizable to a large contingent of patients, including post-ICU patients cared for on the wards, as well as those who have been discharged home.

In addition to their potential use following an ICU admission, wearables may also play a role in monitoring inpatients for signs of clinical deterioration, so as to identify as soon as possible any patient needing a higher level of care. Early Warning Systems (EWS) have been developed to address a “failure to rescue” problem, in which critical illness is identified too late [21]. Wearable devices stand to enhance data collection and monitoring both prior to and following an ICU admission, and as such is of growing importance in critical care research.

Interest in the clinical use of wearable devices and mobile health technology is increasing [2, 22]. While clinical evaluations of this technology remain scarce, some rigorous evaluations have been reported among healthy volunteers [4, 5] and among outpatients [23]. To our knowledge, this is the first study to examine the feasibility of using commercially available wearable devices among hospital inpatients to evaluate for heart rate derangements and sleep quality.

Wearables have the potential to become a useful tool in the early detection of critical illness. Heart rate is factored into the majority of EWS algorithms [24–28], and while the role of an EWS in reducing mortality remains unclear, there is evidence to suggest that these systems may be helpful [24]. Changes in heart rate may also portend changes in clinical status among ICU survivors on the wards or following hospital discharge. In this study, the high specificity but low to moderate sensitivity identified for the detection of tachycardia suggests that as currently configured, wearable-derived heart rate tracking would be highly specific, thereby mitigating alarm fatigue, but may lack sensitivity in some situations, resulting in missed detection of heart rate excursions. Ultimately, further confirmatory studies are required, which should also investigate alternate approaches to event detection, such as those based on proportional changes in heart rate. One potential limitation of wearable-enabled heart rate monitoring is a direct result of the PPG-based sensing mechanism employed, which may perform poorly in patients with a pulse deficit, such as those in atrial fibrillation.

Hospitalized patients often have a severely disrupted sleep, which may impair recovery [12]. Illness, medications, around-the-clock care activities, and environmental light and noise may contribute to perturbed sleep. Consumer-grade wearables with sleep monitoring capabilities could facilitate the routine evaluation of sleep among inpatients and the assessment of sleep-promoting interventions. Resource-intensive polysomnography (PSG) is impractical for routine sleep monitoring, and compliance with sleep questionnaires and sleep diaries is poor among inpatients [29]. Continuous data collection from wearables is passive and unobtrusive, and wearables are far less expensive than both PSG equipment and standard actigraphy devices.

Two recent studies have compared commercial-grade wearables with PSG in healthy subjects [10, 11]. Mantua et al. found a strong correlation in total sleep time between wearable-derived data and PSG, and De Zambotti et al. found good agreement between wearables and PSG in measuring sleep, despite slight but significant overestimation of total sleep by the wearable devices. Altered sleep and activity patterns among inpatients may decrease the accuracy of wearables, which rely on movement to determine wakefulness, and could overestimate sleep in inpatients, who may be awake but immobile for long periods. The wearable device used in our study only counts periods of inactivity that exceed one hour as sleep, and may not capture fragmented naps, which are common in critically ill patients [30, 31].

Our study has a number of limitations that should be considered in interpreting the results. Conclusions regarding the influence of non-sinus rhythm on the accuracy of heart rate monitoring are limited by the relatively low prevalence of this condition in the study cohort, as are the findings relating sleep with delirium, which also had a low prevalence. While we considered the absence of sleep quality measures reported to indicate an absence of sleep during the monitoring period, an alternate interpretation is that these conditions reflect a failure of data capture. It is worth noting, however, that for the cases included that recorded no sleep data, heart rate data was successfully collected, making a failure of data capture an unlikely explanation for these findings. Lastly, differences between the internal clocks of the wearables and bedside monitors may have resulted in asynchronous heart rate recordings being treated as simultaneous, although correction factors were used in the analysis, and the time differences observed were shorter than the 5 min sampling interval of the wearable device.

## Conclusions

In this observational study, we compared heart rate and sleep data recorded from a commercial-grade wearable device, with data from cardiac telemetry and sleep questionnaires. Devices showed high specificity and moderate sensitivity for the detection of tachycardia, with better performance in patients in sinus rhythm. Sleep quality metrics were moderately correlated with questionnaire data.

Future research in this area should focus on improving tachycardia detection, evaluating patients on the wards and at home, integrating wearable-derived data into the study of ICU recovery, and determining the impact of integrating wearable devices into hospital-wide EWS or rapid response services. Patients with arrhythmias should be studied as a subgroup in order to better define the accuracy of wearable-based heart rate sensing in this population. Further validation of sleep quality accuracy using other comparators such as PSG or conventional actigraphy would be useful, as would assessments of the accuracy of activity tracking.

## Additional file

Additional file 1: Details regarding microbiologic assessment of the wearable devices as well as specifics regarding wearble device data capture and analysis. (PDF 49 kb)

## Abbreviations

CAM: Confusion Assessment Method
ECG: Electrocardiography
EWS: Early Warning System
HR: Heart rate
ICU: Intensive care unit
MRSA: Methicillin-resistant *Staphylococcus aureus*
PPG: Photoplethysmography
PSG: Polysomnography
RCSQ: Richards-Campbell Sleep Questionnaire
SPO2-R: Pulse oximetry pulse rate recordings (SPO2-R)
