# Supplementary material for: Use of wearable devices for post-discharge monitoring of ICU patients: a feasibility study
Source: J Intensive Care. 2017 Nov 21;5:64. doi: 10.1186/s40560-017-0261-9 (PMC5698959; doi:10.1186/s40560-017-0261-9)
Supplement: Additional file 1: — Details regarding microbiologic assessment of the wearable devices as well as specifics regarding wearble device data capture and analysis. (PDF 49 kb) [file 40560_2017_261_MOESM1_ESM.pdf]

S1 Appendix

Microbiologic Assessment

Devices were swabbed using a Transystem aerobic/anaerobic swab (Copan Diagnostics Incorporated, Mantua, Italy). The swabs were directly plated onto Blood Agar, Chocolate agar and MacConkey plates (Oxoid Incorporated, Nepean, Canada), as well as Thio broth for 24 hours. All the plates and the Thio broth were incubated at 37^oC for 24 hours. Any growth at 24 hours on the culture plates was identified using Matrix-assisted Laser desorption/ionization – Time of Flight (MALDI-TOF) spectroscopy. Visual turbidity detected at 24 hours in the Thio broth was also planted on Blood Agar, Chocolate Agar and MacConkey agar and further incubated for another 24 hours at 37^oC and then identified by MALDI-TOF. This sampling was done for the WDs immediately after removing them from the patients as well as immediately after they were cleaned using hydrogen peroxide disinfectant wipes.

Data Capture

The study used 6 separate wearable devices (3 size large, 3 size extra-large), each of which was assigned a unique email address and log-in credentials for the Fitbit website. An automated R script was used to download and process wearable data from the Fitbit website. Heart rate data are recorded by the wearable every 5 minutes. To provide a gold standard measurement of heart rate, we recovered data from the ICU bedside monitors using specialized software (BedMasterEX, Excel Medical, Jupiter, FL). Data included heart rate values, as well as heart rate data derived from continuous Spo_2 monitoring (SpO_2-R), both recorded every minute. These data were acquired as XML files and processed using an automated Python script to derive minute-level heart rate data. We synchronized bedside monitor data and wearable data using a correction factor that accounted for the difference between each device's internal clock.
